# Sexual mixing in opposite-sex partnerships in Britain and its implications for STI risk: findings from the third National Survey of Sexual Attitudes and Lifestyles (Natsal-3)

**DOI:** 10.1093/ije/dyy237

**Published:** 2018-12-11

**Authors:** Rebecca S Geary, Andrew J Copas, Pam Sonnenberg, Clare Tanton, Eleanor King, Kyle G Jones, Viktoriya Trifonova, Anne M Johnson, Catherine H Mercer

**Affiliations:** Institute for Global Health, University College London, London, UK

**Keywords:** sexual behaviour, heterosexual, sexual mixing, assortative mixing, disassortative mixing, sexual partners, sexually transmitted infection, survey, population

## Abstract

**Background:**

The extent to which individuals are similar to their sexual partners influences STI-transmission probabilities, yet there is a dearth of empirical data, especially those representative of the population.

**Methods:**

Analyses of data reported by 10 759 sexually active people aged 16–74 y interviewed for a British national probability survey undertaken in 2010–12. Computer-assisted self-interviews asked about partner numbers and characteristics of participants’ three most recent partnerships (MRPs). Opposite-sex MRPs were weighted to represent *all* such partnerships in the past year (*N* = 16 451). Estimates of disassortative age mixing (≥±5-y difference), ethnic mixing (partner of a different ethnic group) and geographical mixing (partner from a different region/country when they first met) were calculated, stratified by gender, age group and partnership status (casual/steady). Multivariable regression examined how these disassortative mixing measures were associated with STI-risk measures: condom use at first sex together at the partnership-level and, at the participant-level, STI-risk perception and reporting STI diagnoses.

**Results:**

Disassortative age mixing occurred in around one-third of opposite-sex partnerships, with men ≥5 y older in most cases, although this proportion varied by participant’s gender and age group. Ethnic mixing occurred less frequently (11.3% of men’s and 8.6% of women’s partnerships) as did geographical mixing (14.1 and 16.3%, respectively). Disassortative mixing was more common among casual vs steady partnerships. Condom use at first sex was less likely in women’s partnerships that were age-disassortative [adjusted odds ratio (AOR): 0.79, 95% confidence interval (CI): 0.69–0.95], whereas men reporting disassortative ethnic mixing were more likely to perceive themselves at STI risk (AOR: 1.76, 95% CI: 1.23–2.52) and report STI diagnoses (AOR: 2.37, 95% CI: 1.22–4.59).

**Conclusions:**

Disassortative mixing, although uncommon among opposite-sex partnerships in Britain, is independently associated with STI risk, warranting consideration in STI-prevention efforts.

**Key Messages**

- Understanding STI risk requires knowledge not only of an individual’s socio-demographic and behavioural characteristics, but also those of their partner(s) and the extent to which these characteristics are similar (‘assortative mixing’) or not (‘disassortative mixing’).
- Unlike previous studies that have tended to focus on the most recent partner, thus underestimating casual partnerships, we weighted national probability survey data on individuals’ three most recent partners to represent all opposite-sex partnerships in the past year.
- In most opposite-sex partnerships in Britain, individuals were similar to their partners as *dis*assortative age mixing (≥±5-y age difference) occurred in approximately one-third of partnerships, whereas *dis*assortative ethnic and geographical mixing were less common (fewer than one in six partnerships).
- Disassortative sexual mixing was associated—albeit weakly—with STI-risk indicators, including after controlling for the number and type of sexual partners.
- STI-prevention efforts may benefit from taking account of sexual mixing patterns as well as individual-level risk factors.

## Background

Sexually transmitted infection (STI) prevalence varies within populations according to individuals’ sexual behaviour, such as the number and timing of sexual partners, the type(s) of sexual practice engaged in, the extent to which condoms are used, as well as socio-demographic characteristics such as gender, age and ethnicity, and access to and use of health-care services.^1–3^ As such, STI-transmission probabilities vary when sex occurs between individuals from population groups with different STI prevalences, e.g. between commercial sex workers and their clients,^4^^,^^5^ which in turn influences the rate at which STIs are spread at a population level.^6^ Understanding the extent to which individuals are similar to their partners is therefore important for improving our understanding of STI transmission, including in parameterizing mathematical models that can inform the design and evaluation of STI-control interventions,^6^ such as England’s National Chlamydia Screening Programme.^7^ Where the degree of assortative mixing (i.e. individuals tending to have sexual partners with characteristics and engaging in behaviours similar to themselves^8^) is high, STI-prevention efforts may be more effective if they focus on those at highest risk. In contrast, where mixing is largely disassortative (i.e. individuals tending to have sexual partners with different characteristics and behaviours to themselves^8^), then more generalized approaches may be more appropriate, e.g. population screening. Between these two extremes lies mixing at random, i.e. people having sex with partners at random such that there is no pattern in how their own characteristics and behaviours relate to those of their partners.

Studies that have sought to empirically investigate sexual mixing using data representative of the population are rare,^9^^,^^10^ with analyses of convenience survey data more common, often collected from sexual health clinic attendees,^11–13^ who are known to report greater STI-risk behaviour than observed in the general population.^2^ One exception analysed data from the Health Survey for England (HSE), a national probability survey of all individuals in randomly selected households.^14^ In its 2010 round, data on sexual behaviour were collected for the first time, permitting analyses of the extent of sexual mixing among 943 heterosexual cohabiting couples.^15^ A positive correlation between individuals and their partners’ characteristics was observed for all except one of the demographic characteristics, health behaviours, and sexual histories examined (current mental illness), with 12 of the 17 considered more than moderately assortative, suggesting high levels of assortativity in cohabiting partnerships.^15^ Whereas the vast majority of the adult population in Britain lives with a partner,^16^ non-cohabiting partnerships—who are not captured by the HSE—correspond to a large proportion of all *partnerships.*^17^ This is an even greater issue for young people, who bear the greatest burden of STIs,^1^^,^^2^ the majority of whom do not live with their sexual partners.^16^ Furthermore, there is evidence that non-cohabiting partnerships are more likely to be disassortative than cohabiting partnerships,^18^ and their often shorter duration^19^ enables higher rates of partner change, meaning that they are important for STI transmission within the population.

Using a previously published method,^20^ this paper aims to build upon earlier analyses^17^ as, in addition to describing the extent of age mixing, it describes the extent of ethnic and geographical mixing among all opposite-sex partnerships in the past year in Britain using data from the third National Survey of Sexual Attitudes and Lifestyles (Natsal-3). It also seeks to examine how sexual mixing relates to STI risk, specifically, condom use, STI-risk perception and history of STI diagnosis/es, and whether any associations remain after adjusting for the confounding effects of the number and types of partners participants reported.

## Methods

### Participants and procedures

Full details of the methods used in Natsal-3 have been reported elsewhere.^16^^,^^21^ Briefly, Natsal-3 involved a multistage, clustered, stratified probability sample design and interviewed 15 162 men and women aged 16–74 y, resident in households in Britain between September 2010 and August 2012. The response rate was 57.7%.^16^^,^^21^ One randomly selected person per household was invited to participate in a face-to-face computer-assisted personal interview (CAPI). More sensitive questions were asked in a computer-assisted self-interview (CASI), including those about participants’ sexual partners (defined as ‘people who have had sex together—whether just once, or a few times, or as regular partners, or as married partners’; having sex together was defined as ‘vaginal, oral and anal sexual intercourse’). Detailed questions about participants’ three (where applicable) most recent partners (MRPs) in the past 5 y were also asked in the CASI. This paper focuses on sexual mixing within opposite-sex partnerships; sexual mixing within men’s same-sex partnerships is the focus of a separate paper.

### Measures

The age difference between a participant and their partner was calculated as the male’s age minus the female’s age. Age differences −5 years or +5 years are referred hereon as disassortative age mixing, as defined in previous studies.^17^^,^^22^ Ethnic group was categorized as White, Asian/Asian British, Black/Black British or other, and disassortative ethnic mixing was defined as the participant and partner being from different groups. However, because of our sample size and the relatively low prevalence of people of non-White ethnicity in the British population,^23^ we do not examine the effect of mixing between particular ethnic groups. Disassortative geographical mixing was defined as the partner living in a different region within the same country or another country when they first met.

### Statistical analysis

We initially restricted analysis for this paper to the 11 340/15 162 (74.8%) of Natsal-3 participants who reported having had at least one opposite-sex partner in the past year to provide a contemporary picture of sexual mixing (4749 men, 6591 women). Because of the paper’s use of data collected by the MRP module, participants were also required to have reported data on at least one opposite-sex MRP with whom sex had occurred in the past year. This reduced the sample size from 11 340 to 10 759 (94.9% of eligible participants; 4490 men, 6269 women) who are described in Supplementary Table 1, available as Supplementary data at *IJE* online.

As partner numbers decrease the more recent the timeframe,^16^ focusing on the past year rather than the past 5 y enabled us to increase the proportion of participants with MRP data available for all the partners they reported having in response to a question in the CASI on partner numbers during this time [question ‘Het1Yr’: ‘Altogether, in the last year, how many (women/men) have you had sexual intercourse with?’ where ‘women’ was asked whether the participant was male and vice versa]. 382/4490 (8.5%) eligible men and 334/6269 (5.3%) eligible women reported more than three opposite-sex partners in the past year^16^ and so the MRP questions were not asked for all their partners. Using the total number of partners reported by all participants in response to ‘Het1Yr’ described above (*N* = 16 451), we estimated that the MRP questions were not asked of 19% of all partnerships in the past year (24% of all men’s and 14% of all women’s partnerships in the past year). These unreported partnerships are typically shorter in duration and less likely to be ongoing compared with the most recent^19^ and so, to minimize this potential bias, we weighted participants’ ≤3 MRPs to represent fourth and higher-order partners if they reported more than three opposite-sex partners using a previously published method.^20^ Briefly, MRPs that were assumed to have ended in the past year were weighted to represent participants’ partnerships that were not captured by the MRP questions, i.e. partnerships that were less recent than the three MRPs. For example, if a participant reported that they had four partners in the year prior to interview, then data on their three MRPs were obtained, including whether each MRP had ended or was ongoing. If two MRPs were ongoing while the third MRP had ended, then the two MRPs were each weighted as one to represent just those partnerships, whereas the third MRP would be assigned a weight of two so that it represented itself and the fourth partner for which no detailed data were collected. If, however, all three MRPs had ended, then they would be weighted equally to represent the fourth partner, i.e. each having a weight of 4/3 = 1.333. These partnership-level weights were applied in addition to participant-level weights used to account for unequal selection probabilities and differential non-response.^21^ Doing so results in weighting the 13 824 opposite-sex partners reported in the MRP module to represent the total 16 451 partnerships reported at Het1Yr. Given the study’s large sample size, *p* < 0.01 is considered as evidence of an association, whereas 0.01 < *p* < 0.05 is considered as weak (or weaker) evidence of an association.

We used Stata (version 14) complex survey analysis functions to incorporate the weighting, stratification of the data^24^ and geographical clustering of participants.^25^ After describing the sample characteristics (Supplementary Table 1, available as Supplementary data at *IJE* online), we used boxplots to show the distribution of age differences between partners by gender and age group, which highlight the medians and inter-quartile ranges (IQRs). We then estimated the prevalence of disassortative age, ethnic and geographical mixing, defined above, by gender, age group and partnership type (Supplementary Table 2, available as Supplementary data at *IJE* online, shows how partnership type was categorized). We used the Pearson chi-square statistic (adapted for complex surveys) to determine whether disassortative sexual mixing varied according to these key socio-demographic variables.

We used multivariable logistic regression to examine how each of the three measures of disassortative sexual mixing was associated with three indicators of STI risk at the partnership-level and the participant-level—outcome 1: condom use at first sex in a partnership (yes/no) as a direct measure of risk behaviour within the partnership; outcome 2: participant’s perception of their STI risk (at risk or not; asked face to face using showcards); and outcome 3: whether the participant reported STI diagnosis/es in the past year (asked in the CASI). In the participant-level models (outcomes 2 and 3), disassortative sexual mixing was captured as the participant reporting disassortative age, ethnic or geographical mixing in any of their ≤3 MRPs, i.e. as a marker of being exposed to disassortative sexual mixing in the past year. Potential confounders in the participant-level models were participant’s current age, whether any of their ≤3 MRPs were casual, the total number of opposite-sex partners reported (past year, i.e. Het1Yr) and, for the models looking at disassortative ethnic mixing as a hypothesized explanatory variable, participant’s ethnicity. In contrast, in the partnership-level models, participant’s age at first sex with the partner was considered as a confounder.^17^Supplementary Table 3, available as Supplementary data at *IJE* online, gives the adjusted odds ratios (AORs) for all variables included in each model. In the partnership-level models, we additionally tested for effect modification by including a term corresponding to the interaction between age and the age mixing indicator. Participant-level models were limited to those 7880 participants aged 16–44 y and partnership-level models were limited to those opposite-sex partners (10 651 unweighted, 11 206 weighted) reported by the 7880 participants aged 16–44 y as STI risk is considerably lower in older people,^1^ although, for completeness, modelling results for those aged 16–74 y are presented in Supplementary Table 4, available as Supplementary data at *IJE* online.

### Ethical approval

The Natsal-3 study was approved by the Oxfordshire Research Ethics Committee A (reference: 09/H0604/27).

## Results

### Sample characteristics

The sample was evenly distributed by age for men and women (Supplementary Table 1, available as Supplementary data at *IJE* online). Among those aged 16–74 y, only a minority—approximately 1 in 10—were of non-White ethnicity. The distribution of opposite-sex partner numbers was highly skewed, with men reporting larger numbers of partners on average than women (18.5% of men reported more than one partner vs 12.5% of women). Men were also more likely to report that at least one of their three MRPs was casual (18.3 vs 11.9% of women).

### Disassortative age mixing

**Figure 1.**
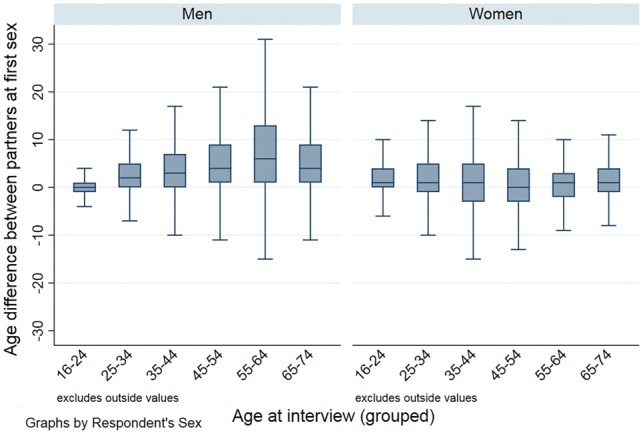
Boxplot of age differences between partners by participant’s gender and age group. Denominator: opposite-sex partners in the past year reported by Natsal-3 participants aged 16–74 y who reported 1+ opposite-sex partners in the past year, data on ≥1 opposite-sex MRPs in the MRP module, and the age of their MRP(s). Unweighted N=13 604 partners (5 867 men’s partners; 7 737 women’s partners); Weighted N=16 201 partners (8 816 men’s partners; 7385 women’s partners).

The median age difference between opposite-sex partners varied slightly by gender: 2 y (IQR: 0, 5 y) in men’s partnerships and 1 y (–1, 4 y) in women’s partnerships, and also with age, especially among men for whom both the median and IQR increased with age (Figure 1). Around one-third of all men’s and women’s partnerships involved an age difference of ≥±5 y, considered as denoting disassortative age mixing (Table 1). Whereas the proportion of men’s partnerships with disassortative age mixing was low (7.9%) at the youngest ages (16–24 y), this proportion increased to around a half for those age 35 and above. A higher proportion (19.4%) of women’s partnerships than men’s at age 16–24 y involved disassortative age mixing. Although this proportion also increased with women’s age, it ‘plateaued’ at a lower level (between 30 and 40%) compared with men. The man was ≥5 y older than the woman in the majority of partnerships with disassortative age mixing (84.6% of men’s and 71.7% of women’s partnerships with disassortative age mixing), although the corresponding percentages for the youngest age group were 96.9% of women vs 25.3% of men. When stratified by partnership type, disassortative age mixing was more common in men’s and women’s casual partnerships than their steady partnerships, with the gap widening between the corresponding proportions with increasing age, especially for women’s partnerships.

**Table 1.** Age mixing in the population of partnerships by gender, age group and partnership type

	Age group																
	16–24		25–34		35–44		45–54		55–64		65–74			All ages (16–74)		All ages (16–44)	
	%	[95% CI]	%	[95% CI]	%	[95% CI]	%	[95% CI]	%	[95% CI]	%	[95% CI]	*p*-value^a^	%	[95% CI]	%	[95% CI]
**Comparing all partnerships by gender and age group**																	
*Men’s partnerships*													<0.001				
Denominators (unweighted, weighted partnerships)	1989, 2318		1671, 2101		805, 1593		685, 1451		528, 1034		286, 448			5964, 8945		4384, 5916	
Same age (<5 y younger/older)	92.1	[89.1, 94.4]	63.2	[56.6, 69.3]	53.5	[49.2, 57.8]	47.7	[41.5, 53.9]	40.4	[35.3, 45.8]	50.0	[43.6, 56.5]		63.1	[60.6, 65.5]	71.7	[68.8, 74.5]
Man ≥5 y younger	5.9	[3.9, 8.9]	6.9	[5.5, 8.5]	6.7	[5.1, 8.6]	4.3	[3.0, 6.2]	3.8	[2.4, 6.0]	4.4	[2.5, 7.8]		5.7	[4.9, 6.6]	6.4	[5.4, 7.7]
Man ≥5 y older	2.0	[1.2, 3.3]	29.9	[23.8, 36.9]	39.9	[35.6, 44.2]	48.0	[41.6, 54.6]	55.8	[50.3, 61.2]	45.6	[39.1, 52.2]		31.2	[28.8, 33.7]	21.8	[19.1, 24.8]
Any disassortative age mixing	7.9		36.8		46.6		52.3		59.6		50.0			36.9		28.2	
% of disassortative where man older	25.3		81.3		85.6		91.8		93.6		91.2			84.6		77.3	
*Women’s partnerships*													<0.001				
Denominators (unweighted, weighted partnerships)	2454, 1823		2622, 1645		1107, 1723		885, 1252		524, 705		240, 317			7832, 7466		6113, 5136	
Same age (<5 y younger/older)	80.6	[77.8, 83.2]	67.2	[64.6, 69.6]	58.4	[54.6, 62.2]	61.9	[58.7, 65.7]	64.2	[59.6, 68.6]	72.7	[66.2, 78.4]		67.5	[65.6, 69.2]	68.9	[66.4, 71.3]
Man ≥5 y younger	0.6	[0.3, 1.3]	6.4	[5.4, 7.6]	12.0	[8.2, 17.2]	17.4	[14.4, 20.8]	17.2	[13.9, 21.0]	8.4	[5.0, 13.7]		9.2	[8.1, 10.5]	6.2	[5.2, 7.5]
Man ≥5 y older	18.8	[16.3, 21.6]	26.5	[24.3, 28.9]	29.6	[23.5, 36.5]	20.7	[17.9, 23.8]	18.6	[15.3, 22.5]	18.9	[14.2, 24.8]		23.3	[21.2, 25.6]	24.8	[22.1, 27.9]
Any disassortative age mixing	19.4		32.9		41.6		38.1		35.8		27.3			32.5		31.0	
% of disassortative where man older	96.9		80.5		71.2		54.3		52.0		69.2			71.7		80.0	
*p*-value for difference between men’s and women’s partnerships	<0.001		0.37		0.007		<0.001		<0.001		<0.001			<0.001		0.24	
**Comparing men’s partnerships by type and age group**																	
*Men’s steady partnerships*													<0.001				
Denominators (unweighted, weighted partnerships)	1086, 1178		1204, 1368		653, 1291		561, 1146		443, 836		263, 406			4210, 6225		2916, 3799	
Same age (<5 y younger/older)	93.2	[87.9, 96.2]	67.4	[60.5, 73.6]	57.2	[52.9, 61.4]	53.2	[48.5, 57.8]	46.2	[40.5, 51.9]	54.0	[47.3, 60.6]		63.7	[61.4, 66.0]	71.8	[68.5, 74.8]
Man ≥5 y younger	4.2	[1.7, 9.8]	6.2	[4.9, 7.9]	6.6	[5.0, 8.8]	4.5	[3.1, 6.6]	3.7	[2.2, 6.0]	4.1	[2.3, 7.3]		5.1	[4.3, 6.2]	5.8	[4.5, 7.3]
Man ≥5 y older	2.6	[1.3, 5.1]	26.4	[20.1, 33.8]	36.2	[32.1, 40.5]	42.3	[37.8, 46.9]	50.2	[44.3, 56.0]	41.9	[35.4, 48.7]		31.1	[28.9, 33.5]	22.5	[19.6 25.7]
Any disassortative age mixing	6.8		32.6		42.8		46.8		53.9		46.0			36.2		28.3	
% of disassortative where man older	38.2		81.0		84.6		90.4		93.1		91.1			85.9		79.5	
*Men’s casual partnerships*													<0.001				
Denominators (unweighted, weighted partnerships)	903, 1140		467, 733		152, 302		124, 305		85, 197		23, 42			1754, 2719		1467, 2116	
Same age (<5 y younger/older)	91.0	[87.0, 93.9]	55.2	[42.2, 67.5]	37.4	[27.0, 49.1]	26.4	[14.3, 43.5]	15.6	[6.5, 32.8]	9.3	[3.4, 22.8]		61.6	[55.7, 67.2]	71.7	[65.9, 76.9]
Man ≥5 y younger	7.7	[5.0, 11.7]	8.1	[5.3, 12.1]	6.8	[3.9, 11.7]	3.4	[1.2, 9.4]	4.2	[1.1, 14.3]	7.5	[1.0, 38.8]		7.0	[5.3, 9.0]	7.7	[5.8, 10.1]
Man ≥5 y older	1.3	[0.7, 2.4]	36.8	[24.8, 50.7]	55.8	[44.0, 67.0]	70.2	[51.8, 83.8]	80.2	[63.3, 90.5]	83.2	[60.2, 94.2]		31.4	[25.8, 37.7]	20.6	[15.6, 26.8]
Any disassortative age mixing	9.0		44.9		62.6		73.6		84.4		90.7			38.4		28.3	
% of disassortative where man older	14.4		82.0		89.1		95.4		95.0		91.7			81.8		72.8	
*p*-value for difference between men’s steady and casual partnerships	0.19		0.13		0.001		0.003		0.002		0.001			0.35		0.41	
**Comparing women’s partnerships by type and age group**																	
*Women’s steady partnerships*													<0.001				
Denominators (unweighted, weighted partnerships)	1650, 1111		2113, 1281		957, 1386		789, 1141		492, 673		231, 307			6232, 5899		4681, 3748	
Same age (<5 y younger/older)	81.7	[78.9, 84.2]	69.8	[67.4, 72.0]	66.5	[59.0, 73.3]	64.7	[60.8, 68.4]	66.0	[61.2, 70.4]	74.5	[68.0, 80.0]		70.1	[68.0, 72.0]	72.1	[69.4, 74.6]
Man ≥5 y younger	0.9	[0.4, 2.0]	5.2	[4.3, 6.4]	11.3	[8.3, 15.2]	13.7	[11.2, 16.7]	14.9	[11.7, 18.7]	6.3	[3.6, 10.7]		8.7	[7.7, 9.8]	6.2	[5.1, 7.5]
Man ≥5 y older	17.4	[15.0, 20.2]	25.0	[22.9, 27.2]	22.2	[18.6, 27.6]	21.6	[18.6, 24.9]	19.2	[15.8, 23.2]	19.2	[14.4, 25.2]		21.3	[19.8, 22.9]	21.7	[19.7, 23.9]
Any disassortative age mixing	18.3		30.2		33.5		35.3		34.1		25.5			30.0		27.9	
% of disassortative where man older	95.1		82.8		66.3		61.2		56.3		75.3			71.0		77.8	
*Women’s casual partnerships*													<0.001				
Denominators (unweighted, weighted partnerships)	804, 712		509, 364		150, 337		96, 112		32, 33		9, 10			1600, 1567		1432, 1387	
Same age (<5 y younger/older)	78.9	[73.3, 83.6]	57.9	[51.3, 64.3]	24.5	[9.6, 49.5]	34.1	[23.2, 46.8]	28.5	[15.3, 46.8]	14.4	[2.7, 50.4]		57.6	[47.1, 67.5]	60.4	[48.2, 71.5]
Man ≥5 y younger	0.1	[0.2, 1.0]	10.4	[7.7, 14.1]	14.9	[5.6, 33.9]	54.8	[41.5, 67.4]	64.3	[45.4, 79.6]	76.6	[37.6, 94.7]		11.4	[8.6, 15.1]	6.3	[4.5, 8.9]
Man ≥5 y older	21.0	[16.3, 26.6]	31.6	[25.6, 38.3]	60.7	[29.2, 85.2]	11.2	[6.1, 19.7]	7.2	[1.8, 25.4]	8.9	[1.0, 47.9]		31.0	[20.3, 44.1]	33.2	[21.7, 47.2]
Any disassortative age mixing	21.1		42.0		75.6		66.0		71.5		85.5			42.4		39.5	
% of disassortative where man older	99.5		75.2		80.3		17.0		10.1		10.4			73.1		84.1	
*p*-value for difference between women’s steady and casual partnerships	0.093		<0.001		0.013		<0.001		<0.001		<0.001			0.068		0.092	

^a^*p*-value for overall association

### Disassortative ethnic mixing

Disassortative ethnic mixing occurred in 11.3% of all men’s and 8.6% of all women’s partnerships, with these proportions declining with age for both genders (Table 2). For both men and women, disassortative ethnic mixing was more common in casual partnerships than steady partnerships (20.6 vs 7.2%, respectively, of men’s partnerships and 14.9 vs 6.9%, respectively, of women’s partnerships).

**Table 2.** Disassortative ethnic and geographic mixing in the population of partnerships by gender, age group and partnership type

	Age group																	
	16–24		25–34		35–44		45–54		55–64		65–74			All ages (16–74)		All ages (16–44)		
	%	[95% CI]	%	[95% CI]	%	[95% CI]	%	[95% CI]	%	[95% CI]	%	[95% CI]	*p*-value^a^	%	[95% CI]	%	[95% CI]	
Ethnic mixing																		
**Comparing all partnerships by gender and age group**																		
*Men’s partnerships*																		
Denominators (unweighted, weighted partnerships)	1990, 2319		1667, 2098		804, 1576		683, 1445		527, 1031		286, 448			5957, 8918		4461, 5993		
Disassortative ethnic mixing	13.5	[10.3, 17.4]	16.4	[11.8, 22.4]	8.6	[6.3, 11.6]	8.4	[5.8, 11.9]	7.6	[4.7, 12.1]	3.4	[1.3, 8.3]	<0.001	11.3	[9.7, 13.2]	13.2	[11.0, 15.8]	
*Women’s partnerships*																		
Denominators (unweighted, weighted partnerships)	2454, 1823		2614, 1641		1105, 1721		883, 1249		523, 705		239, 316			7818, 7454		6173, 5185		
Disassortative ethnic mixing	13.2	[10.8, 16.1]	10.2	[8.6, 12.1]	6.4	[4.3, 9.5]	7.3	[5.5, 9.6]	3.0	[1.9, 4.8]	3.0	[1.6, 5.9]	<0.001	8.6	[7.5, 9.8]	10.0	[8.5, 11.7]	
*p*-value for difference between men’s and women’s partnerships	0.92		0.011		0.25		0.53		0.005		0.83			0.006		0.021		
**Comparing men’s partnerships by type and age group**																		
*Men’s steady partnerships*																		
Denominators (unweighted, weighted partnerships)	1086, 1178		1200, 1364		652, 1275		559, 1140		442, 834		263, 406			4202, 6198		2938, 3817		
Disassortative ethnic mixing	8.5	[6.2, 11.7]	9.2	[7.0, 12.0]	8.2	[5.9, 11.2]	6.9	[4.8, 9.7]	3.5	[2.1, 6.0]	2.2	[1.0, 4.8]	0.003	7.2	[6.3, 8.3]	8.7	[7.3, 10.2]	
*Men’s casual partnerships*																		
Denominators (unweighted, weighted partnerships)	903, 1140		467, 733		151, 301		124, 305		85, 197		22, 42			1753, 2718		1521, 2174		
Disassortative ethnic mixing	18.6	[13.3, 25.3]	29.9	[19.2, 43.4]	10.3	[5.5, 18.7]	14.1	[6.0, 29.9]	24.6	[12.3, 43.1]	15.3	[2.3, 57.9]	0.09	20.6	[16.3, 25.7]	21.2	[16.4, 27.1]	
*p*-value for difference between men’s steady and casual partnerships	0.002		<0.001		0.49		0.11		<0.001		0.031			<0.001		<0.001		
**Comparing women’s partnerships by type and age group**																		
*Women’s steady partnerships*																		
Denominators (unweighted, weighted partnerships)	1649, 1111		2110, 1279		955, 1384		787, 1137		491, 672		230, 306			6222, 5889		4714, 3774		
Disassortative ethnic mixing	11.0	[9.0, 13.3]	7.9	[6.6, 9.5]	6.2	[4.4, 8.7]	6.4	[4.7, 8.7]	2.4	[1.4, 4.1]	2.9	[1.5, 5.8]]	<0.001	6.9	[6.1, 7.8]	8.2	[7.1, 9.4]	
*Women’s casual partnerships*																		
Denominators (unweighted, weighted partnerships)	804, 712		504, 362		150, 337		96, 112		31, 31		9, 10			1594, 1563		1458, 1410		
Disassortative ethnic mixing	16.8	[12.1, 22.9]	18.1	[13.6, 23.8]	7.3	[2.7, 17.9]	16.1	[8.5, 28.5]	12.5	[4.3, 31.3]	6.4	[0.8, 37.9]	0.15	14.9	[11.3, 19.3]	14.9	[11.0, 19.8]	
*p*-value for difference between women’s steady and casual partnerships	0.023		<0.001		0.71		0.008		0.002		0.48			<0.001		<0.001		
Geographical mixing																		
**Comparing all partnerships by gender and age group**																		
*Men’s partnerships*																		
Denominators (unweighted, weighted partnerships)	1964, 2283		1654, 2068		803, 1589		683, 1438		528, 1034		284, 443			5916, 8855		4421, 5941		
Disassortative geographical mixing	10.1	[8.0, 12.6]	13.2	[10.7, 16.1]	19.4	[16.3, 22.9]	14.5	[11.2, 18.5]	16.8	[12.6, 22.0]	12.7	[8.1, 19.4]	<0.001	14.1	[12.8, 15.5]	13.7	[12.2, 15.3]	
*Women’s partnerships*																		
Denominators (unweighted, weighted partnerships)	2441, 1811		2611, 1639		1105, 1721		884, 1252		522, 704		240, 317			7803, 7444		6157, 5171		
Disassortative geographical mixing	10.5	[8.6, 12.8]	15.4	[13.6, 17.4]	27.4	[11.0, 53.6]	13.9	[11.6, 16.6]	12.6	[9.9, 15.9]	10.9	[7.4, 15.9]	0.089	16.3	[11.2, 23.1]	17.7	[10.8, 27.7]	
*p*-value for difference between men’s and women’s partnerships	0.64		0.89		0.037		0.83		0.16		0.96			0.46		0.31		
**Comparing men’s partnerships by type and age group**																		
*Men’s steady partnerships*													<0.001					
Denominators (unweighted, weighted partnerships)	1085, 1178		1203, 1366		653, 1291		561, 1146		443, 836		262, 404			4207, 6221		2941, 3835		
Disassortative geographical mixing	6.8	[5.1, 9.1]	12.5	[10.0, 15.4]	18.7	[15.4, 22.5]	12.9	[9.9, 16.6]	14.0	[10.5, 18.5]	13.1	[8.2, 20.3]		13.0	[11.7, 14.5]	12.8	[11.2, 14.6]	
*Men’s casual partnership*s													0.15					
Denominators (unweighted, weighted partnerships)	878, 1104		451, 702		149, 298		122, 292		85, 197		22, 39			1707, 2632		1478, 2104		
Disassortative geographical mixing	13.6	[10.1, 18.0]	14.6	[10.0, 20.7]	22.4	[14.7, 32.7]	20.7	[10.0, 38.0]	28.4	[14.8, 47.6]	8.6	[2.3, 27.2]		16.7	[13.7, 20.1]	15.2	[12.4, 18.5]	
*p*-value for difference between men’s steady and casual partnerships	<0.001		0.45		0.44		0.22		0.047		0.52			0.026		0.16		
**Comparing women’s partnerships by type and age group**																		
*Women’s steady partnerships*													0.057					
Denominators (unweighted, weighted partnerships)	1649, 1109		2106, 1277		956, 1386		789, 1141		490, 671		231, 307			6221, 5890		4711, 3772		
Disassortative geographical mixing	8.3	[6.7, 10.3]	14.2	[12.3, 16.4]	22.8	[11.3, 40.7]	13.3	[10.9, 16.2]	12.3	[9.5, 15.7]	11.1	[7.4, 16.1]		14.6	[11.1, 19.0]	15.6	[10.5, 22.6]	
*Women’s casual partnerships*													0.077					
Denominators (unweighted, weighted partnerships)	792, 702		505, 362		149, 336		95, 111		31, 32		9, 10			1581, 1552		1446, 1399		
Disassortative geographical mixing	14.0	[10.2, 18.9]	19.7	[15.2, 25.1]	46.6	[13.5, 83.0]	19.7	[11.5, 31.7]	19.0	[8.7, 36.8]	6.4	[0.8, 37.9]		22.8	[12.2, 38.5]	23.3	[11.8, 40.7]	
*p*-value for difference between women’s steady and casual partnerships	0.007		0.036		0.021		0.18		0.29		0.59			0.019		0.015		

^a^*p*-value for overall association.

### Disassortative geographical mixing

The proportion of partnerships involving someone from a different region or a different country was similar for men and women (14.1 and 16.3%, respectively) (Table 2). Fluctuations in disassortative geographical mixing by age group were evident but there was no obvious linear trend. Similar proportions of steady and casual partnerships involved disassortative geographical mixing, apart from at the youngest ages, when this type of mixing was twice as likely in casual vs steady partnerships (13.6 vs 6.8% of men’s partnerships; 14.0 vs 8.3% of women’s partnerships to those aged 16–24 y).

### Disassortative sexual mixing and its implications for STI risk

Focusing on those aged 16–44 y, the age group at greatest risk of STIs,^1^ condoms were less likely to have been used at first sex in women’s partnerships involving disassortative age mixing, including after adjusting for the age of the woman at first sex with her partner [AOR: 0.79, 95% confidence interval (CI): 0.65–0.95] (Table 3). The effect of having an age-disassortative partnership on condom use at first sex was greater the younger the woman was at the start of the partnership. For example, women under 20 y at first sex with their partner had an AOR of 0.56 (95% CI: 0.42–0.75), whereas it was 0.83 (95% CI: 0.64–1.08) for women aged 20 y or older at this time. Furthermore, the AOR for condom use was 0.76 (95% CI: 0.59–0.99) for women’s partnerships where specifically the male partner was ≥5 y *older* but there was no difference in the odds where the male was ≥5 y *younger* (both relative to the woman being of a similar age to her partner). Neither ethnic mixing nor geographical mixing was associated with the partnership-level indicator of STI risk for either gender.

**Table 3.** Associations between disassortative sexual mixing and STI-risk indicators by gender for participants aged 16–44 at interview

	Men's partnerships							Women's partnerships						
	% of partnerships	[95% CI]	Odds ratio	AOR^a^	[95% CI]	*p*-value	Denominator^c^	% of partnerships	[95% CI]	Odds ratio	AOR^a^	[95% CI]	*p*-value	Denominator^b^
Partnership-level outcome: Condom use at first sex with partner														
Hypothesized explanatory variables														
Disassortative age mixing with partner						0.76							0.015	
No (assortative mixing)	60.1	[57.0, 63.2]	1	1	–		2588, 3294	62.9	[58.7, 66.9]	1	1			3598, 3003
Yes (disassortative mixing)	51.4	[44.3, 58.5]	0.70	0.95	[0.67, 1.34]		847, 1316	55.9	[46.9, 64.5]	0.75	0.79	[0.65, 0.95]		1640, 1415
														
Disassortative ethnic mixing with partner						0.53							0.58	
No (assortative mixing)	57.0	[54.1, 59.8]	1	1			3142, 4142	60.8	[55.0, 66.3]	1	1			4776, 4046
Yes (disassortative mixing)	60.7	[48.4, 71.8]	1.17	1.19	[0.69–2.04]		336, 520	58.6	[52.5, 64.4]	0.91	0.91	[0.64–1.28]		497, 399
														
Disassortative geographical mixing with partner						0.91							0.23	
No (assortative mixing)	57.9	[54.7, 60.9]	1	1	–		3031, 4054	58.5	[55.9, 61.1]	1	1	–		4617, 3654
Yes (disassortative mixing)	54.4	[48.2, 60.6]	0.87	0.98	[0.74, 1.31]		442, 602	69.8	[46.7, 85.9]	1.64	1.85	[0.68, 5.04]		651, 785


	Male participants							Female participants						
	% of participants	95% CI	Odds ratio	AOR^a^	95% CI	p-value	Denominator^c^	% of participants	95% CI	Odds ratio	AOR^a^	95% CI	p-value	Denominator^c^
Participant-level outcome: Any STI risk perceived^d^														
Hypothesized explanatory variables														
Disassortative age mixing in any of (max.) 3 MRPs						0.86							0.031	
No, only assortative mixing	35.5	[33.3–37.8]	1	1	–		2246, 2216	25.3	[23.6, 27.1]	1	1	–		3075, 2187
Yes, age mixing in at least one partnership	33.6	[30.2–37.3]	0.92	0.98	[0.79, 1.22]		914, 1055	31.4	[28.8, 34.1]	1.35	1.21	[1.02, 1.44]		1639, 1103
														
Disassortative ethnic mixing in any of (max.) 3 MRPs						0.002							0.49	
No, only assortative mixing	32.5	[30.5, 34.6]	1	1	–		2780, 2897	25.8	[24.3, 27.3]	1	1	–		4218, 2954
Yes, ethnic mixing in at least one partnership	53.1	[47.2–58.9]	2.35	1.76	[1.23, 2.52]		380, 375	41.2	[35.7, 46.9]	2.02	1.11	[0.82, 1.49]		496, 336
														
Disassortative geographical mixing (when first met) in any of (max.) 3 MRPs						0.43							0.33	
No, only assortative mixing	34.1	[32.0, 36.3]	1	1	–		2646, 2700	26.3	[24.8, 28.0]	1	1	–		3984, 2772
Yes, geographical mixing in at least one partnership	38.6	[33.7, 43.8]	1.21	1.11	[0.86, 1.43]		514, 572	32.8	[28.8, 37.0]	1.37	1.12	[0.89, 1.42]		730, 519
Participant-level outcome: STI diagnosis in the past year														
Hypothesized explanatory variables														
Disassortative age mixing in any of (max.) 3 MRPs						0.55							0.054	
No, only assortative mixing	1.4	[1.0, 2.0]	1	1	–		2233, 2197	1.5	[1.0–2.1]	1	1	–		3062, 2181
Yes, age mixing in at least one partnership	1.6	[0.9, 2.6]	1.10	1.23	[0.63, 2.40]		906, 1047	2.1	[1.5–3.0]	1.46	1.64	[0.99, 2.72]		1637, 1102
Disassortative ethnic mixing in any of (max.) 3 MRPs						0.011							0.12	
No, only assortative mixing	1.2	[0.8, 1.6]	1	1	–		2762, 2873	1.4	[1.1, 1.9]	1	1			4203, 2946
Yes, ethnic mixing in at least one partnership	4.0	[2.3, 6.7]	3.53	2.37	[1.22, 4.59]		377, 372	4.4	[2.5, 7.5]	3.23	1.83	[0.86, 3.89]		496, 336
Disassortative geographical mixing (when first met) in any of (max.) 3 MRPs						0.87							0.062	
No, only assortative mixing	1.5	[1.1, 2.0]	1	1	–		2632, 2678	1.5	[1.2, 2.0]	1	1	–		3969, 2764
Yes, geographical mixing in at least one partnership	1.5	[0.7, 3.1]	1.02	0.94	[0.43, 2.04]		507, 567	2.8	[1.7, 4.5]	1.86	1.68	[0.98, 2.90]		730, 519


^a^AOR, adjusted odds ratio; for partnership-level outcome (condom use at first sex with partner), OR-adjusted for participant’s age at first sex with that partner; for participant-level outcomes (STI-risk perception and reported STI diagnosis), ORs adjusted for participant’s age at interview, whether any of their (max.) 3 MRPs were casual, participant’s opposite-sex partner numbers in the past year and participant’s ethnicity when the sexual mixing indicator related to ethnic mixing.

^b^Unweighted, weighted partnerships in the past year of sexually active participants aged 16–44.

^c^Unweighted, weighted participants, defined as those sexually active and aged 16–44.

^d^Participants were asked: ‘What do you think about the risks to you, personally, with your present lifestyle of getting a sexually transmitted infection that is not HIV?’ Response options were: greatly at risk; quite a lot; not very much; not at all. For the purposes of these (binary) analyses, this was coded as ‘not at all at risk’ vs ‘at some risk’.

At a participant-level, there was some weak evidence that women—but not men—who had at least one MRP involving disassortative age mixing were more likely to perceive themselves to be at risk of STIs (AOR: 1.21, 95% CI: 1.02–1.44). There was stronger evidence that men—but not women—who had at least one MRP involving disassortative ethnic mixing were more likely to perceive themselves to be at risk of STIs (AOR: 1.76, 95% CI: 1.23–2.52). Disassortative geographical mixing was not associated with STI-risk perception among men or women. There was weak evidence of an association for men between ethnic mixing and reporting STI diagnosis/es in the past year (AOR: 2.37, 95% CI: 1.22–4.59). When all three sexual mixing indicators were included in the models, similar effect sizes were observed as for when each sexual mixing indicator was considered in the model by itself (data not shown).

## Discussion

In Britain, people tend to be similar to their opposite-sex partners in terms of their age and ethnic group, and to be from the same geographical region when they meet. Around one-third of all partnerships involved disassortative age mixing, whereas smaller proportions of partnerships—less than one-sixth—entailed disassortative ethnic and/or geographical mixing. Disassortative sexual mixing is more common in men’s partnerships than women’s, which may in part reflect how a larger proportion of men’s reported partnerships are casual, as disassortative sexual mixing was often more prevalent in casual than steady partnerships. We found disassortative sexual mixing to be associated with markers of STI risk independently of key confounders including age and the number and type(s) of partners reported. Of note, the odds of condom use at first sex with a partner were lower for women in age-disassortative partnerships, whereas the odds of men perceiving themselves to be at risk of STIs and/or reported STI diagnoses were higher if they had had recent ethnic-disassortative partnerships. However, we observed no evidence of a consistent relationship between sexual mixing and STI-risk markers, and the effect sizes observed were weaker than those for more established risk factors, such as age and number and type(s) of partner, which we adjusted for in our models (see Supplementary Table 4, available as Supplementary data at *IJE* online). This may reflect how sexual mixing is more complex conceptually and to measure than factors such as age and partner numbers, in part because it corresponds to not just the individual’s characteristics, but also those of their partners and their sexual network more broadly.

A key strength of this study is that we analysed probability survey data, meaning that our findings can be considered as broadly representative of the British general population.^21^ Whereas the response rate was 57.7%, this is consistent with other major social surveys undertaken contemporaneously in Britain^26^^,^^27^ and the co-operation rate was 65.8% (of all contacted addresses known to be eligible).^28^ Nonetheless, we acknowledge that non-response could be a source of bias for our data. We aimed to minimize this bias by weighting the sample so that it was broadly representative of the underlying population with respect to the distribution of the sexes, age and regions as used in the census.^21^ Furthermore, the sampling strategy used for the Natsal studies means that the target population is specifically the population resident in private households in Britain and, as such, excludes individuals living in institutions, whose behaviour could differ from others’. Whereas this sampling strategy is also a potential source of bias, the institutionalized population constitutes a relatively small proportion of the British population.^21^ We also endeavoured to make our sample of partnerships broadly representative of the population of opposite-sex *partnerships* experienced by people living in Britain in the past year. This involved taking account not just of the current or most recent partner, as in many other studies,^29–32^ but also weighting the three MRPs that were asked about in detail in Natsal-3 to represent less recent partnerships for whom this information was not collected.^20^ As such, we were able to account for under-represented partnerships, which are more likely to be casual, shorter in duration^19^ and involve greater disassortative sexual mixing^18^—characteristics that may facilitate STI transmission.

Whereas Natsal-3 collected a wealth of data from participants on their sexual behaviour, it has relatively limited data on their partners’ characteristics and specifically only three socio-demographic variables. However, it is worth noting that one study that had data on socio-demographic as well as sexual risk assortativity showed that both are likely to be important determinants of STI transmission.^18^ Furthermore, our paper builds upon analyses of Natsal-2 data^17^ as, in addition to disassortative age mixing, it investigated the extent of disassortative ethnic and geographical mixing and their significance for STI transmission. However, their relatively low population prevalence meant that we had to use relatively crude binary variables and were limited in the extent to which we could discriminate between categories. For example, limited numbers of partnerships where one or both partners were of an ethnicity other than ‘White’ meant that we were obliged to use broad ethnic categories, including a ‘Black/Black British’ group despite epidemiological differences in STI prevalence between Black Caribbean and Black African populations.^33^ Similarly, we used a crude measure of disassortative geographical mixing, as this applies to partnerships formed between people from different regions within the same country as well as partnerships involving people from different countries and global regions. This is a particular limitation when considering how disassortative geographical mixing is associated with STI risk, as STI prevalence, and thus transmission probabilities, varies considerably globally.^34^ With all socio-demographic mixing indicators, we acknowledge that these characteristics must work through the more proximate behavioural or biological determinants to impact on STI transmission. In addition, whereas we included both partnership- and participant-level markers of STI risk, we recognize that our participant-level measures of sexual mixing correspond to any mixing in participants’ ≤3 MRPs, which may not correspond to the partnership(s) in which STI(s) were acquired or led to the participant perceiving themselves at risk of STIs. Furthermore, we recognize that, by analysing cross-sectional survey data, it is not possible to determine the chronology of events, e.g. whether having an age-disassortative partnership occurred before or after an STI diagnosis, nor assume causality more broadly.

Focusing on partnerships in the past year meant that all partners in this timeframe were captured in our analyses for a very high proportion of study participants. Whereas those reporting STI diagnosis/es were more likely to be those who reported larger numbers of partners than asked about in the MRP module,^1^ we used multivariable models to adjust for the total number of partners in the past year. By taking into account this established driver of STI transmission in our analyses, we have importantly demonstrated an independent, although weak, relationship between disassortative age and ethnic mixing and STI risk.

The predominance for men—rather than women—to be ≥5 y older in the majority of opposite-sex partnerships involving disassortative age mixing is consistent with previous studies in and outside of Britain.^15^^,^^18^^,^^35^ However, this was not observed for women in casual partnerships in whom the proportion with a male partner who was ≥5 y *younger* increased with age, although the denominator (the number of casual partnerships among women in older age groups) was small in absolute terms and also relative to their male counterparts, which in itself is a notable gender difference. These age mixing patterns and differences in the extent of ethnic and geographical mixing may reflect the availability of eligible partners in an individual’s socio-sexual network,^36^ as well as social norms, e.g. regarding what constitutes an appropriately aged partner, which may be less stringent for casual partnerships.^37^

Disassortative age mixing has been shown to manifest itself, especially at younger ages, in gender power imbalances,^29–31^ resulting e.g. in condoms being less likely to be used, as we and others have found.^38^^,^^39^ However, it was not possible to determine from the quantitative data Natsal-3 collected whether such power imbalances were the reason for non-use of condoms. It is also worth noting that our measure of condom use only corresponds to the first occasion of sex with the partner, so does not capture how well condoms were used on that occasion, nor consistency of use during the partnership. Indeed, evidence suggests that condom use quickly wanes, on average within 3 weeks of first sex together.^40^ Regarding our measure of STI-risk perception, only a single question was used and this may not capture the complexity of this concept, e.g. individuals’ risk perception may change over time and people may perceive their risk to be different for different STIs. Furthermore, the placement of this question—after the CASI’s detailed questions about sexual behaviour—may have influenced participants’ assessment of their risk. Also, as it was asked in the face-to-face section of the interview, albeit using a showcard so that participants only needed to give a response code, it is plausible that responses may still be subject to social-desirability bias.

In addition to differential misreporting by gender,^41^ the observed gender differences in the three measures of sexual mixing reflect how men’s and women’s partners are not from closed populations.^42^ For example, men are more likely than women to report new sexual partners from outside of the UK,^16^ thus partners who are ineligible themselves to participate in Natsal-3; they are also more likely to report paying for sex and so these partners are less likely to be captured by a survey like Natsal.^42^ However, in contrast to analyses of the previous Natsal in which the target population was men and women aged 16–44 y,^17^ these latest analyses of Natsal-3 data correspond to people from a much wider age range (16–74 y) meaning that they capture a greater proportion of both male and female partners, as well as the adult population, thus providing a more complete picture of age mixing in the population. Nonetheless, the age mixing patterns we observed are similar to those from our earlier analyses,^17^ suggesting that this component of partnerships has remained constant between 2000 and 2010, despite evidence of individual-level behaviour changing over time.^16^ Finally, and as in our earlier paper,^17^ we were not able to reliably quantify the extent to which the observed amount of disassortative mixing differs from that which would be expected if mixing occurred at random in the population. This relates in part to crudely categorizing ethnicity, differences in how participants may interpret ‘region’ and also challenges in defining the appropriate population from which to calculate expected numbers. Under mixing at random, most partnerships would involve disassortative age mixing, since, for any individual, most potential partners across the full age range of 16–74 y would be ≥±5 y different in age. Our findings therefore indicate that the pattern of mixing in Britain in terms of age is strongly assortative, and likewise for geographical mixing too. Although we also found that only a minority of partnerships involve disassortative ethnic mixing, this is more difficult to interpret. Because 86% of the British population are estimated to be of White ethnicity,^23^ this relative homogeneity ensures that the majority of partnerships will be ethnically-assortative whatever the underlying pattern of mixing. In terms of the role of ethnically-disassortative partnerships in STI transmission, we, like others,^12^^,^^43^ found that men who had had such partnerships were more likely to report STI diagnoses, but this was not observed for women.

Future research needs to focus on improving understanding the granularities of disassortative mixing, especially given its potential significance in STI transmission. Population-based studies therefore need to be adequately powered, including for ethnic minority groups. It is also vital that studies ask participants about more than just their most recent or cohabiting partner and, whereas it is unlikely to be feasible to ask all participants about all partners, the resulting data should be weighted to take account of those partnerships that are not asked about, which are more likely to be casual. Stratifying analyses by partnership type is also necessary to avoid masking differences in mixing. Future studies should also seek to obtain data on partners’ sexual behaviour, especially their partner’s partner numbers given its importance in STI transmission.^1^ Ideally, this is obtained directly from the partners—as in HSE-2010^15^—as there is evidence that individuals have a poor ability to assess their partners’ behaviour, especially that of non-cohabiting partners.^44^

In terms of implications for policy and practice, the evidence that sexual partnerships in Britain are mainly assortative suggests that STI-prevention efforts are best focused on those individuals at highest risk. However, as there was some evidence that disassortative mixing was independently associated with markers of STI risk, it is worth considering, from a public health perspective, whether highlighting disassortative sexual mixing in health-promotion messaging might be helpful e.g. for identifying those most likely to benefit from STI testing. Similarly, asking patients about their ‘exposure’ to disassortative sexual partnerships may warrant investigation to ascertain its predictive value and utility in clinical triage in contrast, or in addition, to conventional risk-assessment questions such as those on partner numbers and/or condom use. Qualitative research has shown that people are generally willing to answer questions about their partners, at least in the context of a survey,^45^ but further research would be needed to assess the social acceptability in a clinical context of ‘labelling’, e.g. having an older partner, as an STI risk. Even if this is not the case, these data show that STI-prevention efforts may benefit from taking account of sexual mixing patterns as well as individual-level risk behaviour.

## Funding

Natsal-3 was supported by grants from the Medical Research Council (G0701757) and the Wellcome Trust (084840), with contributions from the Economic and Social Research Council and Department of Health. The sponsors of the study had no role in study design, data collection, data analysis, data interpretation or writing of the report. The corresponding author had full access to all the data in the study and had final responsibility for the decision to submit for publication.

**Conflict of interest:** A.M.J. has been a Governor of the Wellcome Trust since 2011. The other authors declare that they have no conflicts of interest.

## Supplementary Material

Supplementary Tables
